# Long non-coding RNA SND1-IT1 accelerates cell proliferation, invasion and migration via regulating miR-132-3p/SMAD2 axis in retinoblastoma

**DOI:** 10.1080/21655979.2021.1909962

**Published:** 2021-04-12

**Authors:** Dong-Fang Yin, Xue-Jun Zhou, Na Li, Hui-Jie Liu, Hu Yuan

**Affiliations:** a Medical Department of Ophthalmology, Chinese PLA General Hospital, Beijing, China; b Medical Department of Otolaryngology, Head and Neck Surgery, Chinese PLA General Hospital, Beijing, China

**Keywords:** LncRNA SND1-IT1, miR-132-3p, SMAD2, biomarker, retinoblastoma, metastasis

## Abstract

Long noncoding RNAs (lncRNAs) have been identified as prognostic biomarkers and functional regulators in human tumors. In our study, we aim to investigate the roles of lncRNA SND1-IT1 (SND1-IT1) in retinoblastoma (RB). We observed that SND1-IT1 was highly expressed in both RB specimens and cells, and associated with poorer prognosis of RB patients. Functional investigation revealed that downregulation of SND1-IT1 suppressed RB cell proliferation, migration and invasion *in vitro* and restrained RB tumorigenesis in vivo. MiR-132-3p was predicted to interact with SND1-IT1. RT-qPCR and dual-luciferase reporter assays verified the regulation of miR-132-3p by SND1-IT1 in RB cells. In addition, SND1-IT1 enhanced the expression of SMAD2 by sponging miR-132-3p. Rescue experiments revealed that knockdown of miR-132-3p reversed the inhibiting effects of miR-132-3p knockdown on RB cells. Overall, SND1-IT1 can promote the progression of RB cells through miR-132-3p/SMAD2 axis, suggesting that l SND1-IT1 might be a novel biomarker and potential target for RB.

## Graphical abstract

**Figure:**
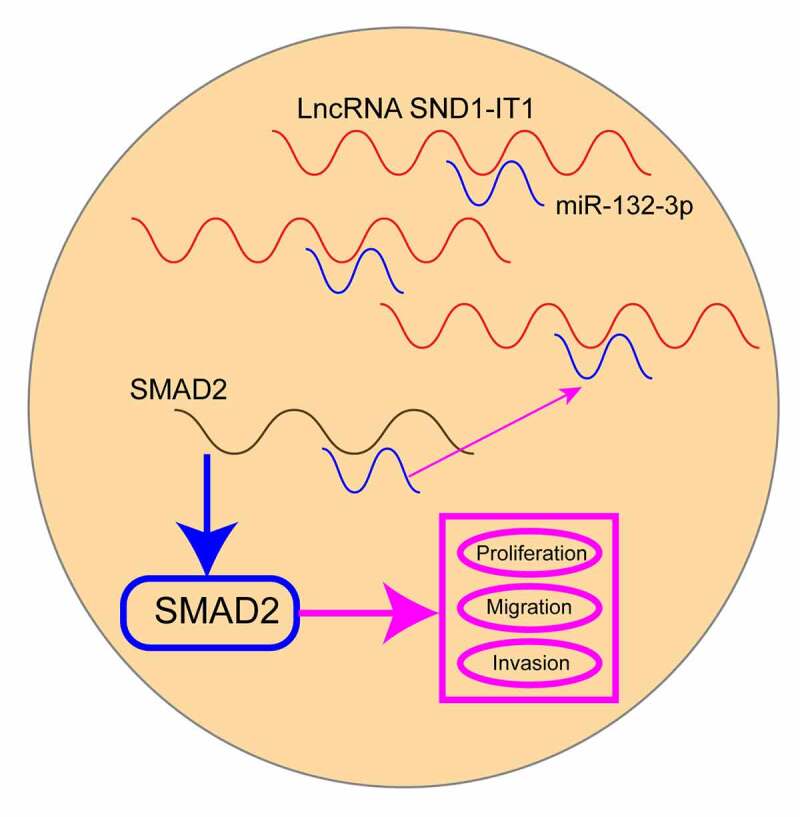

## Introduction

Retinoblastoma (RB) refers to an infrequent childhood carcinoma of the retina and may be lethal if untreated, taking up 2–4% of all tumors with malignancy in children aged less than 6 years [1,2]. It is very likely to result in loss of eye or vision. It is estimated that approximately 1% of all deaths among infants and young children were involved in this disease [3]. Despite the multidisciplinary synthetic treatments, such as chemotherapy, radiotherapy and surgical resection, cases with recurrent or metastatic RB still have an extremely poor outcome [4,5]. Thus, the vital molecule of growing, invading and migrating procedures pertaining to RB cells should be clarified.

Long noncoding RNAs (lncRNAs) refer to not coding RNAs, greater than 200 nucleotides long, without proteins encoding [6]. LncRNAs have been proved to fulfill extensive works (e.g., chromatin modifying, cell destiny determining, RNA alternative splice and genomic imprinting) [7]. Over the past few years, lncRNA has been reported to facilitate and suppress several procedures in carcinoma processes (e.g., cell proliferating process and programmed cell death, cell migrating and invading processes) [8,9]. To be specific, lncRNA FTX has been suggested facilitating the proliferating and migrating processes of gastric carcinoma cells through sponging miRNA-144/ZFX axis [10]. Overexpression of lncRNA DANCR resulted in the promotion of cellular ability in osteosarcoma through modulating miRNA-216a-5p [11]. Up to date, many functional lncRNAs have been demonstrated in many types of tumors. However, only a few lncRNAs have been functionally identified in RB.

LncRNA SND1 intronic transcript 1(SND1-IT1) was a newly identified lncRNA which was firstly reported to exhibit a low level in laryngeal squamous cell carcinoma specimens based on microarray datasets [12]. However, its overexpression in osteosarcoma was reported [13]. Up to date, because of the limited report of the expressing pattern of SND1-IT1 in different types of tumors, the potential function of SND1-IT1 in tumors was hard to judge in this moment. In this study, we aimed to explore whether SND1-IT1 was abnormally expressed in RB, and further study its potential function and clinical significance in RB patients.

## Materials and methods

### Cases and tissue samples

On the whole, 98 RB tissues and 58 adjacent common retina tissue specimens originated in RB cases having undergone enucleation surgery at the Chinese PLA General Hospital. Before the collection of tissue samples, all cases received no tumor-striking treating processes and surgery preoperatively. The authors acquired overall clinical samples in the diagnosing process in advance to the tumor-striking therapeutic process and then conducted the preservation using liquid nitrogen. All patients received written informed consent and the study protocol was approved by the Ethical Committee of the Chinese PLA General Hospital (Approval No.: CPGH20191034).

### Cell culture and transfection

The American Tissue Culture Collection provided human RB cell lines (Y79, SO-RB50 and WERI-RB1) and the retinal pigment epithelial cell line of human (ARPE-19). All cells underwent the culturing process in a routine manner in RPMI-1640 system (Invitrogen, Shenzhen, Guangzhou, China) at 37°C with 5% CO_2_. For supplementing the culturing system, the study employed antibiotics and 10% fetal bovine serum (FBS; Qishan Technology, Haidian, Beijing, China). To silence SND1-IT1 expressions, two individual siRNAs (si-SND1-IT1-1 and si-SND1-IT1-2) and an extra negative control siRNA (si-NC) received the development and optimization with GenePharma (Pudong, Shanghai, China). The sequences of si-SND1-IT1 were shown as follows: si-SND1-IT1-1 (sense, 5 -CCCUAGCUACCCACUCGGGGCAC-3; and antisense, 5 -CCCGUCCUGGACCCAUCGCAAU-3); si-SND1-IT1-2 (sense, 5;-CAGGCCCUGCAUUUGCGUACGCA-3 ; and antisense, 5;-CCCTGCACCACUUUCGUCCACA-3;). The duplicate short hairpin RNAs in terms of SND1-IT1 (sh-SND1-IT1), miR-132-3p mimics, NC mimics, miR-132-3p inhibitors and miR-NC were also produced by Genepharm. With the use of Lipofectamine® 2000 reagent (Thermo Fisher Scientific, Inc., Haidian, Beijing, China), cell transfections were carried out. The efficient property of transfecting process received the detection using RT-qPCR or western blotting experimentally processes.

### RNA extraction and qRT-PCR analysis

Based on TRIzol reagent (Invitrogen, Carlsbad, CA, USA), overall RNA received the extraction in tissues or cells under culture. RevertAid First-Strand cDNA Synthesizing kit (Thermo Scientific) was employed to achieve the reverse transcribing process. RT-PCR was performed using ABI Prism 7900HT (Applied Biosystems, Guangzhou, Guangdong, China) following the reagents’ orientation. GAPDH and U6 became inner standard using 2^−ΔΔCt^ approach. Table 1 lists the sequences of the primers.

**Table 1.** The primers used in this study for RT-PCR

Names	Sequences (5ʹ-3ʹ)
SND1-IT1: F	ACGCCAGCACATCTGCTGCAC
SND1-IT1: R	GTCGAACGGTCCAGCTCAC
miR-132-3p: F	GCGCGCGTAACAGTCTACAGC
miR-132-3p: R	GTCGTATCCAGTGCAGGGTCC
SMAD2: F	CGTCCATCTTGCCATTCACG
SMAD2: R	CTCAAGCTCATCTAATCGTCCTG
GAPDH: F	GGAGCGAGATCCCTCCAAAAT
GAPDH: R	GGCTGTTGTCATACTTCTCATGG
U6: F	ATTGGAACGATACAGAGAAGATT
U6: R	GGAACGCTTCACGAATTTG

### CCK-8 assays

This study determined the influence of SND1-IT1 on cell viable property by CCK-8 assays. In brief, cells underwent the plating process based on 96-well plates under 3000 cells/well density. In terms of each well, the authors introduced 90 μl of system and 10 μl of CCK-8. The authors maintained the dish in the incubating element using 5% CO_2_ at 37^◦^C for 1–2 h. This study determined absorbance (optical density) at 450 nm under a microplate reading element (Molecular Devices, Sunnyvale, CA, USA).

### Colony formation assays

This study conducted colony-forming experimental processes for detecting the clone forming capability and comparing the diversification in the groups of WERI-RB1 and SO-RB50 cells, respectively. A certain number of cells under transfection underwent the seeding process to six-well plates and the 10-day incubation. The 15 min fixing process using 4% formaldehyde (Boinqi, Chengdu, Sichuan, China) was carried out, and the staining process was conducted using 0.1% crystal violet (Solarbio, Pudong, Shanghai, China) for 5 min. This study counted colonies over 1 mm in diameter.

### Transwell assays

4 × 10^4^ transfected WERI-RB1 and SO-RB50 cells under the resuspension in 200 μL serum-free system received the seeding process in the above transwell chamber precoated with Matrigel (BD Biosciences, Guangzhou, Guangdong, China). Cells received the maintaining process in the system with no serum in the above chamber, and the system covering 10% FBS received the introduction to be chemoattractant in the down chamber. When 24 h incubating process was achieved, cells not migrating or invading via the film received the elimination. When 3–4 h incubating process was achieved, the cells under the migration on the low surface received the fixing and staining process using 0.5% crystal violet. The authors carried out the counting process of 6 random fields in the respective well.

### Wound healing assays

Transfected WERI-RB1 and SO-RB50 cells underwent the culturing process and stopped under over 90% confluence. Next, cell layers underwent the scratching process by employing a plastic scribing element. The wound received the observation after 24 h as well as the imaging process based on microscopy. In terms of the migrating rate, cell coverage fraction across the line received the measurement.

### Ethynyldeoxyuridine (EdU) assay

The cell proliferating process received the assessment with an EdU experimental kit (RiboBio, Haidian, Beijing, China) by complying with the manufacturer’s instructions. Cells underwent the seeding process with 96-well plates under 5 × 10^3^ density cells per well when the transfecting process was achieved. Next, 50 μM EdU labeling system received the introduction to the cells. Cell nucleus underwent DAPI staining under darkness, as well as the observing process by microscopy (Thermo Fisher Scientific).

### Tumor formation assay in vivo

The Chinese PLA General Hospital provided nude mice aged 4 weeks. About 6.0 × 106 cells underwent the inoculation in the right flank pertaining to the respective nude mouse based on subcutaneous injecting process. This study measured the subcutaneous tumor volumes per 7 days by employing caliper for 28 days, which was obtained to be a × b^2^ × 0.5 (a, maximal diameter; b, minimal diameter). The mice received the sacrifice at 3–4 weeks after injection, and the tumors received the weighing process. All animals-related experimental processes gained the approval from the Ethics Committee of Chinese PLA General Hospital.

### Subcellular distribution

Based on a PARIS Kit (Life Technologies, Shenzhen, Guangdong, China), nuclear RNA and cytoplasmic received the extracting process. qRT-RCR was used to determine the overall RNA. Internal references were GAPDH and U6 in terms of the cytoplasm and nucleus, separately.

### RNA pull-down experimental processes

Based on Nuclear Protein Extraction Kit (Thermo Scientific Fermentas), this study achieved WERI-RB1 and SO-RB50 cells’ nuclear extracts. SND1-IT1 RNA received the biotin-labeling process by Shenggong Co., Ltd (Shenzhen, Guangzhou, China). Next, this study introduced Pierce streptavidin-agarose beads (150 μl) to the mentioned mix. Next, the reacting processes received the incubation for 35 min, and then the binding RNAs underwent the elution. Lastly, based on qPCR, this study determined eluted miR-132-3p levels.

### Dual luciferase reporter assays

This study performed the luciferase reportion element experimental processes following the previous description. The wild and mutant reporting element vector of SND1-IT1 (pmirGLO-SND1-IT1-wt or pmirGLO- SND1-IT1-mut) and SMAD2 (pmirGLO-SMAD2-wt or pmirGLO-SMAD2-mut), covering assessed wild or mutant miR-132-3p binding site, underwent the synthesis based on GenePharma. If SO-RB50 and WERI-RB1 cells exhibited 70% confluence, the synthesized reporter plasmids were co-transfected with miR-132-3p mimics or mimic control, respectively, by Lipofectamine 2000 (Invitrogen, Pudong, Shanghai, China). Forty-eight hours after transfection, the authors carried out the luciferase activity experimental process based on the Dual-Luciferase Reporter Assay System (Promega, Chengdu, Sichuan, China).

### Western blot assays

Total protein was extracted from the cells using RIPA lysis buffer (CWBIO, Pudong, Shanghai, China) and quantified with a Protein BCA Assay Kit (Bio-Rad, Hangzhou, Zhejiang, China). Equal amounts of proteins were separated by 10% SDS-PAGE, tank-based transferred to nitrocellulose membrane, and blocked for 0.5 h by TBS, followed by the incubation with the appropriate primary antibody. After washed in TBST (Yunshan Technology, Shenzhen, Guangdong, China), the membranes were further incubated with a horseradish peroxidase-conjugated secondary antibody (1:2000) for 1 h at room temperature. The proteins were detected by chemiluminescence. Primary and secondary antibodies were purchased from Lika Technology (Hangzhou, Zhejiang, China).

### Statistical analysis

Overall statistical analyzing processes were conducted based on SPSS 18.0 software (SPSS Inc., Chicago, IL, USA). To draw a comparing process between two groups, the authors employed a two-tailed Student’s t testing process. Many groups were compared based on one- or two-way ANOVA. Receiver operating characteristic (ROC) curves were set for assessing the diagnostic data of SND1-IT1 in terms of the differentiation of tumors in control groups. The survival curves were determined by the Kaplan-Meier approach, and the diversifications were assessed based on the log-rank testing process. The parameters were employed in multivariate assays by employing the Cox proportional hazards method. P < 0.05 exhibited statistical significance.

## Results

### SND1-IT1 expression is frequently increased in RB

**Figure 1.**
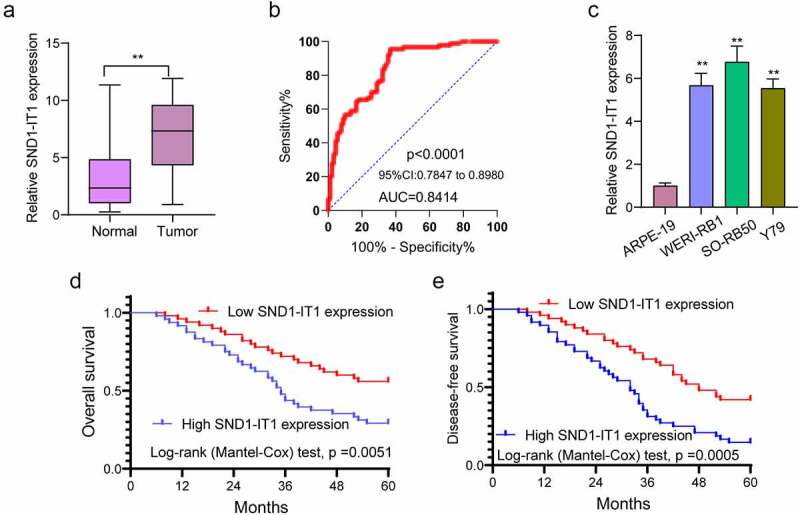
The expression of SND1-IT1 in RB and its clinical significance. (a) SND1-IT1 expression in RB specimens and normal retina tissues was measured using RT-PCR. (b) ROC curves of SND1-IT1 expression for differentiating RB tissues from normal tissue. (c) Relative expression of SND1-IT1 in WERI-RB1, SO-RB50, Y79 and ARPE-19 cells. (d,e) Overall survival rate and disease-free survival rate were calculated using the Kaplan-Meier analysis. **p < 0.01, *p < 0.05

To study whether SND1-IT1 expression was abnormal in RB, we performed RT-PCR to examine its levels in RB specimens and non-tumor retina tissues. As presented in Figure 1(a), we observed that SND1-IT1 expression was distinctly increased in RB specimens compared to non-tumor retina tissues (p < 0.01). According to the ROC assays, our group found that high SND1-IT1 expressing state achieved an AUC value of 0.8414 (95% CI: 0.7847 to 0.8980) for RB (Figure 1(b)). The sensitive and specific properties exhibited by SND1-IT1 expressing states for distinguishing RB samples from normal retina samples were 69.68%/83.29%. In addition, greater states of SND1-IT1 were also identified in three RB cell lines than ARPE-19 (Figure 1(c)). Our findings suggested SND1-IT1 as a novel functional modulator in RB progression.

### SND1-IT1 up-regulation associated with poor prognosis of RB cases

For a better understanding of the clinical relevance of SND1-IT1 expression in RB patients, our group divided the 98 RB patients into a high expression group (n = 48) and a low expression group (n = 50), according to the median expression level of SND1-IT1 (7.53) in all RB samples. Then, we performed chi-square test, finding that high SND1-IT1 expressing state showed a relationship to tumor size (p = 0.007), choroidal invasion (p = 0.036) and optic nerve invasion (p = 0.003)(Table 2). For an in-depth study on the prognostic value of SND1-IT1 expression in RB cases, we collected five-year survival data and performed Kaplan-Meier methods which revealed that cases with high expression of SND1-IT1 had shorter overall survival (OS, p < 0.051; Figure 1(d)) and disease-free survival(DFS, p = 0.005; Figure 1(e)) as compared with the SND1-IT1-low group. More importantly, Multivariate analyses confirmed that SND1-IT1 played a significant role of independent prognostic markers in overall survival (HR = 2.986, 95% CI: 1.238–4.548, p = 0.013) and DFS (HR = 3.218, 95% CI: 1.354–4.896, p = 0.008) of RB cases (Table 3).

**Table 2.** Correlations between lncRNA SND1-IT1 and clinicopathological characteristics in retinoblastoma

Variable	Number	SND1-IT1 expression		*p* value
		High	Low	
Age (years)				0.697
<14	53	25	28	
≥14	45	23	22	
Gender				0.213
Male	55	30	25	
Female	43	18	25	
Size				0.007
<10 mm	54	25	39	
≥10 mm	44	23	11	
Choroidal invasion				0.036
Absent	67	28	39	
Present	31	20	11	
Optic nerve invasion				0.003
Absent	67	26	41	
Present	31	22	9	
Laterality				0.260
Unilateral	34	14	20	
Bilateral	64	34	30

**Table 3.** Multivariate analyses of prognostic factors in RB patients

Variables	Overall survival			Disease-free survival		
	HR	95% CI	*p*-value	HR	95% CI	*p*-value
Age	0.893	0.345–1.563	0.432	1.032	0.445–1.344	0.332
Gender	1.228	0.541–1.957	0.391	1.135	0.663–2.231	0.267
Size	3.213	1.345–4.783	0.007	3.432	1.455–5.212	0.002
Choroidal invasion	2.862	1.324–4.387	0.014	2.953	1.452–4.785	0.009
Optic nerve invasion	3.237	1.486–6.478	0.001	3.663	1.568–7.894	0.001
Laterality	1.238	0.548–2.238	0.238	1.428	0.673–2.103	0.339
SND1-IT1 expression	2.986	1.238–4.548	0.013	3.218	1.354–4.896	0.008

### SND1-IT1 promotes RB cell viability and mobility

**Figure 2.**
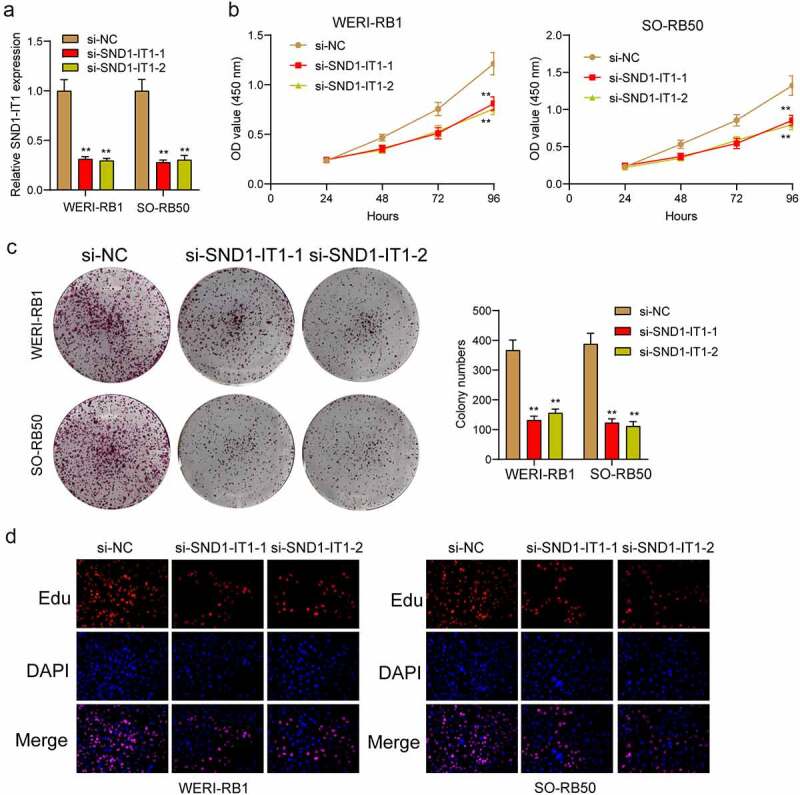
SND1-IT1 promoted proliferation of RB cells. (a) qRT-PCR analysis of the effect on inhibition of si-SND1-IT1 transfection in WERI-RB1 and SO-RB50 cell lines. (b) Cell proliferation was determined by CCK-8 assays. (c) Clone formation assays was used to detect cell proliferation in RB cells. (d) EdU immunofluorescence staining assays for WERI-RB1 and SO-RB50 cells. **p < 0.01, *p < 0.05

**Figure 3.**
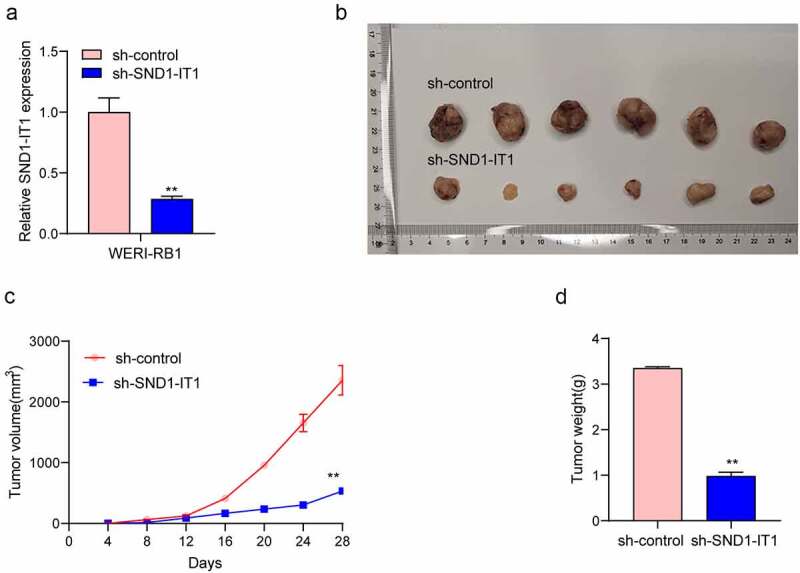
SND1-IT1 accelerated in vivo tumor growth. (a) The downregulation of SND1-IT1 expression in sh-SND1-IT1 transfected WERI-RB1 cells was confirmed by RT-PCR. (b) Tumors derived from mice in two different groups were presented. (c) Tumor volumes were detected every 7 days. (d) The subcutaneous tumor weights were detected at the 28th day after injection. **p < 0.01, *p < 0.05

**Figure 4.**
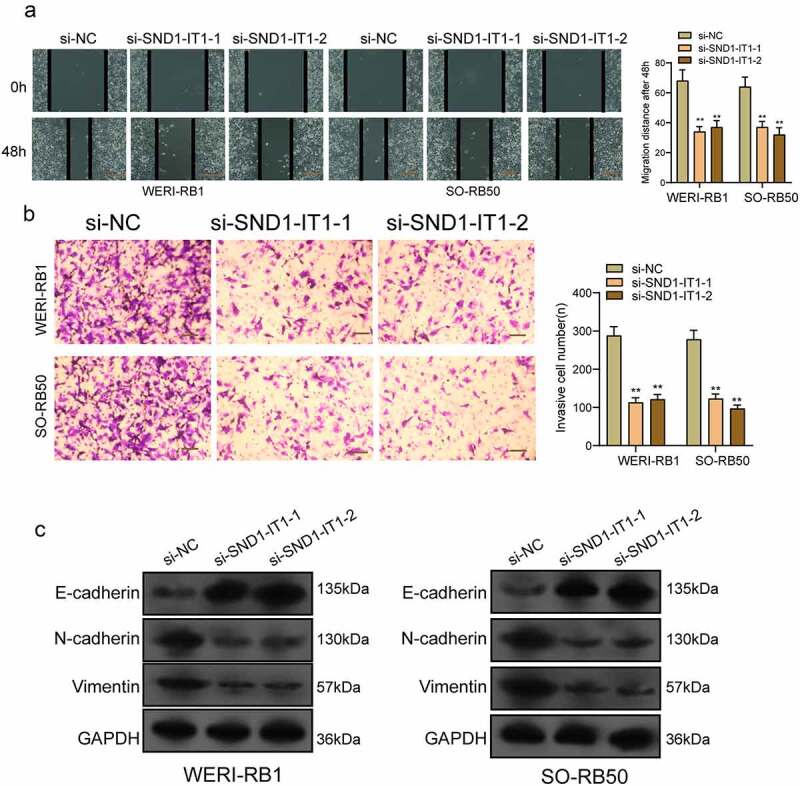
Knockdown of SND1-IT1 suppressed the metastasis of RB cells. (a) Wound healing assay was used to assess the migration of WERI-RB1 and SO-RB50 cells transfected with si-SND1-IT1-1, si-SND1-IT1-2 or si-NC, respectively. (b) The effect of SND1-IT1 on WERI-RB1 and SO-RB50 invasion was determined by transwell assays. (c) Knockdown of SND1-IT1 inhibited the EMT progress. **p < 0.01, *p < 0.05

For determining the effects of SND1-IT1 on RB cell viability and mobility, WERI-RB1 and SO-RB50 cells were transfected with SND1-IT1 siRNAs (si-SND1-IT1-1 and si-SND1-IT1-2). Figure 2(a) confirmed the knockdown efficiencies of ND1-IT1 siRNAs in RB cells by RT-PCR. CCK-8 and colony formation analysis revealed that the viability and clonogenic ability of WERI-RB1 and SO-RB50 cells were distinctly suppressed in the si-SND1-IT1-transfected groups (Figure 2(b,c)). EdU assays also confirmed the suppressor effects of SND1-IT1 knockdown on cell proliferation (Figure 2(d)). Moreover, the results of Xenografts model suggested that the tumor growth speed was slower on nude mice when the subcutaneous injecting process was conducted with sh-SND1-IT1 than the control (Figure 3(a,b)). In addition, our group also observed that the tumor volume and weight underwent the obvious lessening process in sh-SND1-IT1 group compared with control group (Figure 3(c,d)). On the other hand, we also explored the influence of SND1-IT1 on metastasis ability of RB cells. The results of wound healing assay and transwell assays revealed that SND1-IT1 depletion suppressed WERI-RB1 and SO-RB50 cell migration and invasion (Figure 4(a,b)). Given that epithelial-mesenchymal transition (EMT) contributed to tumor metastasis, we further explored the effects of SND1-IT1 on EMT-related markers. According to western blot assays, SND1-IT1 down-regulation was capable of up-regulating E-cadherin and down-regulating Vimentin and N-cadherin (Figure 4(c)). Overall, our findings indicated that SND1-IT1 promoted the progression of RB cells.

### SND1-IT1 acts as a sponge of miR-132-3p in RB cells

**Figure 5.**
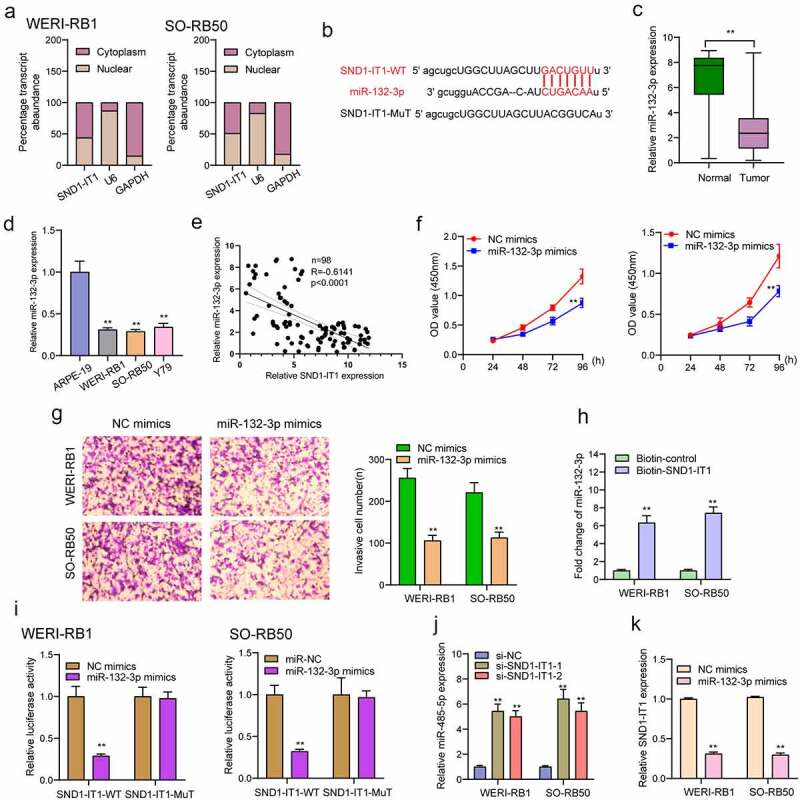
SND1-IT1 acts as a sponge for miR-132-3p. (a) Relative SND1-IT1 expression levels in nuclear and cytosolic fractions of WERI-RB1 and SO-RB50 cells. Nuclear controls: U6; Cytosolic controls: GAPDH. (b) The predicted binding sites of miR-132-3p to the SND1-IT1 sequence. (c) The levels of miR-132-3p in our cohort by RT-PCR. (d) SND1-IT1 expressions in three RB cell lines and ARPE-19 as determined by qRT-PCR. (e) The correlation between miR-132-3p and SND1-IT1 expression analyzed in 98 paired RB samples. (f) CCK-8 determined cell proliferation. (g) Transwell assay was used to detect cell invasion in WERI-RB1 and SO-RB50 cells after overexpression of miR-132-3p. (h) Detection of miR-132-3p using qRT-PCR in the sample pulled down by biotinylated SND1-IT1 probe. (i) Co-transfection of miR-132-3p and SND1-IT1-Wt strongly decreased the luciferase activity. (j,k) The regulatory effects between SND1-IT1 expression and miR-132-3p expression were determined by RT-PCR. **p < 0.01, *p < 0.05

According to existing researches, the main forms of action of lncRNAs include RNA-binding protein interactions or miRNAs as miRNAs for regulating the expressing states of downstream target genes [14]. It has been demonstrated that cytoplasmic lncRNAs are competing endogenous RNAs (ceRNAs) by competitively binding miRNAs [15]. We performed subcellular fractionation, finding that the expression of SND1-IT1 was mainly concentrated in the cytoplasm (Figure 5(a)). Then, we used the bio-informatic tools (Starbase 3.0) to search for the potential miRNAs that can be regulated by SND1-IT1, to our interest, miR-132-3p, which has been demonstrated to be decreased in many types of tumors, could bind to SND1-IT1 (Figure 5(b)) [16]. RT-PCR also showed that miR-132-3p expression was decreased in RB specimens and cell lines (Figure 5(c,d)). Moreover, according to the correlation study here, SND1-IT1 expression is negatively associated with miR-132-3p in 98 RB specimens (Figure 5(e)). Functionally, we showed that overexpression of miR-132-3p suppressed the proliferation and invasion of RB cells (Figure 5(f,g)). To further verify whether miR-132-3p was enrichment in SND1-IT1, we performed a pull-down assay, finding that miR-132-3p was much richer in the precipitate of SND1-IT1 probe than that of in NC probe (Figure 5(h)). More importantly, as indicated from luciferase reporter experimental process, miR-132-3p mimics significantly reduced the luciferase activity of SND1-IT1-WT, whereas it failed to alter the relative luciferase activity of SND1-IT1-MUT (Figure 5(i)). Furthermore, qRT-PCR demonstrated that knockdown of SND1-IT1 distinctly increased miR-132-3p expression in RB cells, and miR-132-3p mimics suppressed SND1-IT1 expressions (Figure 5(j,k)).

### SND1-IT1 regulates SMAD2, a target gene of miR-132-3p

**Figure 6.**
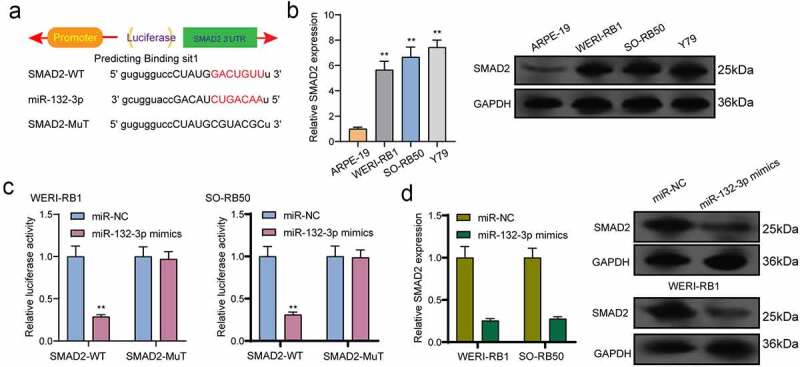
SMAD2 acted as a target of miR-132-3p. (a) The sequences of SMAD2 and miR-132-3p within SMAD2 3ʹ-UTR, including wild-type and mutant. (b) the mRNA and protein levels of SMAD2 were increased in RB cells. (c) Relative luciferase activity of WERI-RB1 and SO-RB50 cells after co-transfection with wild type (wt) or mutant (mut) SMAD2 3ʹ-UTR reporter genes and miR-132-3p mimics. (d) Overexpression of miR-132-3p suppressed the expression of SMAD2. **p < 0.01, *p < 0.05

**Figure 7.**
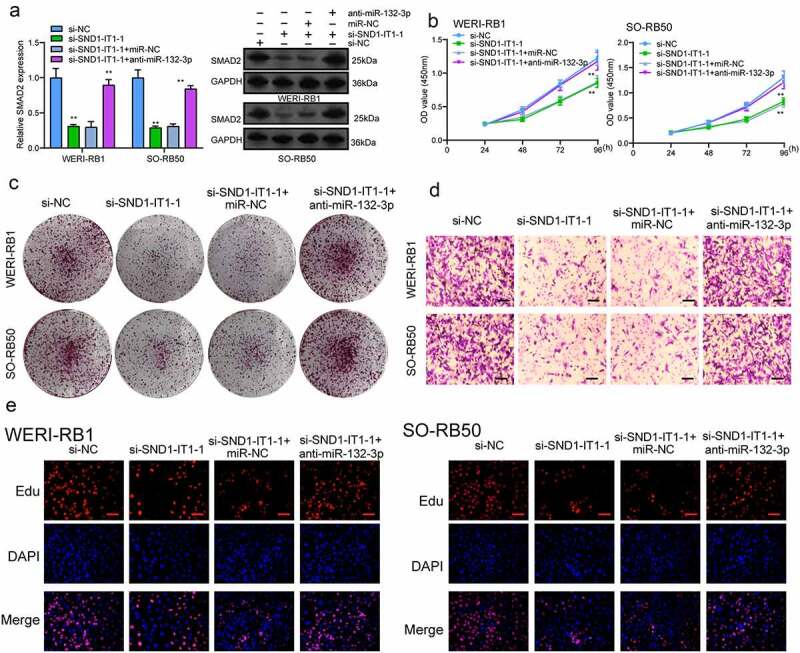
**Knockdown of miR-132-3p attenuates the regulatory effects of SND1-IT1 knockdown on the progression of RB cells**. (a) The expression levels of SMAD2 in WERI-RB1 and SO-RB50 cells after knockdown of SND1-IT1 and/or inhibition of miR-132-3p. The CCK-8 assays (b), colony formation assays (c), Cell invasion(d), Edu assays (e) following knockdown of SND1-IT1 and/or inhibition of miR-132-3p. *p < 0.05, **p < 0.01

For the exploration of the probable effect exerted by miR-132-3p in RB progression, target genes of miR-132-3p underwent the screening process based on bioinformatics predicting process. SMAD2 was identified and further analyzed (Figure 6(a)). Then, we performed Western blot to determine the expression of SMAD2 in RB cells and the results revealed that the mRNA and protein levels of SMAD2 received a remarkable promotion in three RB cells compared to ARPE-19 cells (Figure 6(b)). Further luciferase activity assays demonstrated that the co-transfecting process of miR-132-3p with WT-3ʹUTR in WERI-RB1 and SO-RB50 cells displayed distinct decreased luciferase activity in contrast to the control vector group, while Mut-3ʹUTR luciferase activity remained unchanged (Figure 6(c)). In addition, overexpression of miR-132-3p resulted in a decreased level of SMAD2 at both mRNA and protein levels (Figure 6(d)). To further explore whether miR-132-3p and SMAD2 are responsible for SND1-IT1-mediated effects on RB cells, we performed rescue experiments. As shown in Figure 7(a), we observed that SMAD2 was remarkably down-regulated by SND1-IT1 knockdown in WERI-RB1 and SO-RB50 cells, which could be reversed by the knockdown of miR-132-3p. Besides, the proliferating, migrating and invading suppressions attributed to silencing SND1-IT1 were distinctly attenuated by miR-132-3p exhaustion in WERI-RB1 and SO-RB50 cells2(Figure 7(b–e)). Taken together, our findings suggested SND1-IT1 may contribute to RB progressing process via altering miR-132-3p/SMAD2.

## Discussion

RB is one retinal tumor that is initiated in response to genetic/epigenetic changes culminating in the development of tumors. Up to date, for most RB cases, the five-year survivals remained poor [17,18]. The early detection of RB and the prediction of clinical outcomes before clinical treatments could provide better treatments for RB cases [19]. The application of imaging methods has been reported to display several limits. Hence, the identification of novel diagnosing methods might lead to a more effective treating process for RB cases. In recent years, growing studies have suggested the great potential of lncRNAs used as novel diagnostic and prognostic biomarkers for RB cases [20,21]. Here, a novel RB-related lncRNA, SND1-IT1, was found, which is likely to become a novel biomarker for RB cases. We provided evidence that SND1-IT1 expression was distinctly increased in RB specimens and cell lines. ROC assays demonstrated the diagnostic value of SND1-IT1 in screening RB specimens from non-tumor specimens. Clinical assays revealed that high SND1-IT1 expression was associated with tumor size, choroidal invasion, optic nerve invasion and poor prognosis. Overall, our findings revealed SND1-IT1 as a novel regulator involved in the clinical progression of RB.

In recent years, more and more studies have reported the involvements of lncRNAs in RB progression [22]. For instance, lncRNA THOR, an overexpressed lncRNA in RB, was shown to promote the proliferation and invasion of RB cells via enhancing the combination of c-myc and IGF2BP1 [21]. LncRNA UCA1, a widely studied lncRNA which has been frequently reported to be involved in the progression of various types of tumors, was reported to exhibit a high level in RB. In functional experiments, lncRNA UCA1 was demonstrated to proliferation and multidrug resistance of RB cells via sponging miRNA-513a-5p [23]. These findings highlighted the important effects of lncRNA on RB progression. In recently, lncRNA SND1-IT1 was firstly reported to be highly expressed in osteosarcoma, and its knockdown suppressed the proliferation and metastasis of RB cells via modulating miRNA-665/POU2F1, suggesting SND1-IT1 as a tumor promotor in osteosarcoma [13]. However, its function in other tumors has not been investigated. In this study, we performed loss-of-function assays, finding that knockdown of SND1-IT1 suppressed the proliferation, migration, invasion and EMT progress of RB cells. In addition, in vivo experiments also confirmed that SND1-IT1 down-regulation suppressed xenograft tumor growth in vivo. Our findings were consistent with the tumor-promotive effects of SND1-IT1 on osteosarcoma progression.

Although the regulatory effects of lncRNAs in cellular ability have been frequently demonstrated, the underlying molecular mechanisms by which SND1-IT1 exhibited its tumor-related functions remained largely unclear. Then, we planned to identify the localization of SND1-IT1 in RB cells because lncRNAs functions are dependent on its subcellular localization. It has been confirmed that cytosolic lncRNAs can modulate mRNA stability, protein localization and act as microRNA sponge [24]. Using subcellular fractionation, we observed that a larger proportion of SND1-IT1 was expressed in the cytoplasm, suggesting SND1-IT1 may act as competing endogenous RNAs (ceRNAs) through competitively binding miRNAs. The results of StarBase v3.0 indicated miR-132-3p as a potential target of SND1-IT1. By the use of a series of functional experiments, we observed that miR-132-3p was lowly expressed in RB specimens and cells, and its overexpression suppressed the proliferation and invasion of RB cells. Previously, miR-132-3p has been reported to serve as a tumor suppressor in several types of tumors, including RB. Importantly, the results of RNA-pull down and luciferase activity assays confirmed the direct binding relationship between SND1-IT1 and miR-132-3p. Moreover, the positively regulator effects between SND1-IT1 and miR-132-3p were also confirmed using RT-PCR. Overall, together with the fact that miR-132-3p served as a tumor promotor in RB, SND1-IT1 may display its tumor-promotive roles via sponging miR-132-3p.

SMAD family member 2(SMAD2), located on 18q21.1, is a key component of TGF-β signal transduction, and the suppression of SMAD2 has been observed to result in the inhibition of tumorigenesis, EMT progress and cellular invasion induced by TGF-β pathways [25,26]. Previously, SMAD2 has been reported to be highly expressed in retinoblastoma and its overexpression facilitated the proliferating and metastatic processes of RB cells [27]. In this study, we found that SMAD2 was a potential target of miR-132-3p. This study found overexpression of miR-132-3p suppressing the expression of SMAD2. To further explore whether SND1-IT1 exhibited its function via sponging miR-132-3p/SMAD2 axis, the authors carried out rescue experimental processes, finding that the proliferation, migration and invasion suppression caused by silencing SND1-IT1 were distinctly attenuated by miR-132-3p exhaustion in WERI-RB1 and SO-RB50 cells. Our findings suggested that SND1-IT1 promotes RB progression by targeting miR-132-3p/SMAD2 axis.

## Conclusions

Our study identifies SND1-IT1 as a novel RB-related lncRNA which competitively sponges miR-132-3p to block the suppression influence exerted by miR-132-3p on SMAD2 and then contributes to the proliferating and metastatic processes exhibited by RB cells. In addition, overexpression of SND1-IT1 was closely associated with an aggressive tumor phenotype and adverse prognosis in RB cases. These findings suggest that SND1-IT1 may serve as a potential therapeutic target and a novel biomarker to accurately treat RB.
